# Parental separation and school dropout in adolescence

**DOI:** 10.1177/14034948231164692

**Published:** 2023-04-12

**Authors:** Kateryna Karhina, Tormod Bøe, Mari Hysing, Kristin G. Askeland, Sondre A. Nilsen

**Affiliations:** 1 Department of Psychosocial Science, Faculty of Psychology, University of Bergen, Norway; 2 Regional Centre for Child and Youth Mental Health and Child Welfare, NORCE Norwegian Research Centre, Norway; 3 Department of Health Promotion and Development, Faculty of Psychology, University of Bergen, Norway

**Keywords:** Parental divorce, parental separation, adolescence, school dropout, school completion, upper secondary school

## Abstract

**Aims:**

To document the association between parental separation and school dropout in adolescence and to examine the factors that may potentially account for this association.

**Methods:**

Data stem from the large youth@hordaland study that was linked to the Norwegian National Educational Database to obtain objective measures of educational outcomes and disposable income (*N* = 8323). Logistic regression analysis was used to investigate the association between parental separation and school dropout. A Fairlie post-regression decomposition was used to examine the influence of parental education, household income, health complaints, family cohesion, and peer problems in explaining the association between parental separation and school dropout.

**Results:**

Parental separation was associated with a higher odds ratio (OR) of school dropout in crude and adjusted (adjusted odds ratio (AOR)) analyses (OR=2.16, 95% confidence interval (CI) =1.90–2.45; AOR = 1.72, 95% CI = 1.50–2.00). About 31% of the higher odds of school dropout among adolescents with separated parents was explained by the covariates. The decomposition analysis suggested that parental education (43%) and disposable income (20%) accounted for most of the explained differences in school dropout.

**Conclusions:**

**Adolescents with separated parents are at higher risk for not completing secondary education. Parental education and disposable income accounted for most of the explained differences in school dropout between the groups. Still, the majority of the difference in school dropout remained unaccounted for, indicating that the link between parental separation and school dropout is complex and likely influenced by multiple factors.**

## Background

Children and adolescents with separated parents are at a higher risk of maladjustment across a multitude of outcomes compared with their peers in nuclear two-parent families [1]. The link between parental separation and poorer academic outcomes in adolescence may be particularly important, as it may initiate future disadvantages including occupational and economic instability and poorer health [2].

The association between parental separation and academic achievement among adolescents has been documented in several international studies [2]. For instance, youth with separated parents have been found to have lower test scores and to fail more classes [3] and to have a lower probability of obtaining a university degree than peers in nuclear two-parent families [4]. Parental separation has also been linked to school outcomes in Norway, including higher rates of problems in school [5] and a lower probability of transitioning from lower to higher secondary education [6]. Drawing on the same data as the present study, we have also previously found that adolescents with separated parents have lower grade point average (GPA) than peers in nuclear two-parent families and that GPA varies across post-separation living arrangements [7,8].

The degree to which parental separation is associated with school dropout during adolescence is less explored. Drawing on data from the General Social Surveys from the USA, a study found that living in a single-mother household (compared with nuclear household) caused by divorce at age 16 years was associated with lower odds (odds ratio (OR) = 0.74) of completing high school [9]. Similarly, another study from the USA found that both male (OR = 0.61) and female (OR = 0.66) adolescents experiencing parental divorce had lower odds of completing high school, after accounting for sociodemographic characteristics including maternal education [10]. However, given the differences in social welfare and school systems, the degree to which findings from the USA generalize to the Norwegian context is uncertain.

Several perspectives have been advanced in explaining why parental separation is linked to adverse outcomes among youth [11]. In simple terms, these perspectives focus on factors that may influence the likelihood of parental separation and negative child outcomes (i.e. selection mechanisms), or changes that are set into motion by the process of parental separation.

Socioeconomic status is positively associated with achievement among youth [12]. Higher parental education may influence adolescents’ academic achievement directly through the genetic transfer of academic abilities (e.g. see Pokropek and Sikora [13]) and through parenting practices that foster academic abilities [14]. Parental income may further influence adolescents indirectly by environmental opportunities, such as access to buying books or living in neighbourhoods with better schools [15]. Lower educated couples have a higher risk of divorce than higher educated couples in Norway [16]. Thus, the higher risk for school dropout among youth with separated parents may partly stem from the fact that they tend to have lower educated parents. Although low income may increase the risk of separation, a separation may also lead to reduced economic resources [17]. Accounting for parental education and income is thus important when considering the link between parental separation and school dropout in adolescence.

An association between parental separation and school dropout may also be linked to family processes. Previous work has indicated that youth with separated parents report more family conflicts and lower levels of family cohesion [18]. As family cohesion is positively associated with higher academic achievement and lower probability of school dropout among adolescents [19], such family factors may explain part of the link between parental separation and school dropout in adolescence.

The link between parental separation and school dropout could also be influenced by more proximal factors relating to child adjustment. Health complaints are one potential candidate, as youth with separated parents report more health complaints than peers with non-separated parents [20], and health complaints are robustly associated with a higher risk of school dropout in adolescence [21]. Peer problems is another potential pathway, as adolescents struggling to create bonds with their peers are more likely to drop out from school [22] and there are some indications that children with separated parents have more peer problems than peers in nuclear two-parent families [23]. The link between parental separation and peer problems appears, however, to be poorly documented in adolescence, and there is a need for future work to assess whether peer problems influence the association between parental separation and school dropout.

## Aims

Based on the above considerations, this study sought to document the association between parental separation and school dropout among Norwegian adolescents, by drawing on a large survey of a well-defined cohort of Norwegian adolescents that was linked to objective registry information about school dropout. Another aim was to examine the relative contribution of parental education and income, family cohesion, health complaints and peer problems in explaining the potential association between parental separation and school dropout.

## Methods

### Participants and procedure

This study draws on data from the youth@hordaland study, a population-based survey of adolescents aged 16–19 years conducted in 2012 in Hordaland County, Norway. All adolescents born between 1993 and 1995 were invited to participate. Information about the study was given by email and one school hour was allocated to complete the survey by answering an electronic questionnaire. For those in school, a teacher organized the data collection and ensured confidentiality. For those not at school at the time of the study, the questionnaire could be completed at their convenience during the study period (e.g. at home). Some schools also arranged catch-up days. Efforts were also made for the participation of adolescents in hospitals or institutions. A reminder to complete the questionnaire was sent via email, and by text message to adolescents enrolled in school, and via post to those not attending school.

For the entire study, 10,257 agreed to participate, yielding a response rate of 53%. The current study draws on a subsample of adolescents (*n* = 9166) that consented to register linkage. As the aim of the study was to examine parental separation, adolescents stating that their parents were not living together but that they had not divorced or separated (*n* = 301) were excluded, as the reasons for why their parents were not living together were unknown (e.g. could be owing to death, sickness or that they had never lived together). We also excluded participants with missing information on variables used to define parental separation (*n* = 505, missing = 5.51%), yielding a sample size of *N* = 8360. Further, 37 more were excluded due to missing information on the covariates.

### Ethical permission

The study was approved by the Regional Committee for Medical and Health Research Ethics in the Western Norway (2011/811/REK Vest and SIKT 545201). Following the regulations from the REK and Norwegian health authorities, adolescents aged 16 years and older can make their own decisions regarding their health, and thus consented to participate in the study and to the linkage to the registries. Their parents/guardians were informed of the study.

### Measurements from registers

#### Sex

Sex (female, male) was derived through the personal identity number from the Norwegian National Registry.

#### School dropout

Information on school dropout was obtained from the National Education Database (NUDB, which contains educational statistics and is administered by Statistics Norway. Completion of upper secondary school was defined according to the national definition used by Statistics Norway as graduation within five years from the beginning of the upper secondary school for students enrolled in general tracks and within six years for students enrolled in vocational tracks [24]. Participants who had not completed upper secondary school within five/six years were defined as non-completers.

#### Parental education

Parental education was obtained from NUDB when the adolescents were 16 years old. In the present study, we use a combined measure representing the highest completed educational level in the family. The categories were created in line with the International Standard Classification of Education, resulting in a four-level variable: (1) both parents have no qualifications higher than lower secondary education (ISCED 0–2), (2) at least one parent had qualifications equivalent to ISCED 3–5 (upper secondary education, post-secondary non-tertiary education, short-cycle tertiary education), at least one parent had education of Bachelor’s level or equivalent (ISCED 6), and at least one parent had attained a Master’s or Doctoral level of education (ISCED 7–8). In addition, a separate category indicates parents whose education is not known.

#### Equivalized disposable income (EDI)

We used the EDI from 2011, corresponding to the school year they were in at the time of participating in the youth@hordaland study. EDI is a measure of household income (i.e. the sum of wages and salaries, income from self-employment, property income, and transfers received minus total assessed taxes and negative transfers) that is adjusted by an equivalence scale to account for the notion that households of different compositions may have different economic needs. We use the European Union scale where the first adult is given a weight of 1, subsequent adults are given a weight of 0.5 and each child <14 years is given the weight 0.3 [25]. The logarithmic function was applied to EDI prior to analysis to lower the skewness.

### Measurements from the youth@hordaland study

#### Parental separation

Parental separation was defined according to the adolescents’ answer to the questions ‘Do your parents live together?’ and ‘Have your parents divorced or separated?’ Adolescents confirming that their biological parents lived together were defined as living in a non-separated family, whereas youth reporting that their parents did not live together and that they had divorced or separated were defined as having separated parents [7].

#### Health complaints

Health complaints were measured by five items measuring the frequency of headache, abdominal pain, back pain, neck and shoulder pain, and dizziness during the last six months [26]. Symptoms were rated on a five-point scale from ‘seldom or never’ to ‘about every day’. An overall measure was created by adding the scores together, yielding a sum score ranging from 0 to 20, as supported by a previous study [26].

#### Family cohesion

Family cohesion was measured by the family cohesion subscale of the Resilience Scale for Adolescents (READ) [27]. The factor structure and psychometric properties of the READ have previously been tested and found adequate in a previous study from the youth@hordaland study [28]. In this study, we used the slightly modified version of the family cohesion subscale that was found to have good psychometric properties on the youth@hordaland sample. In this adaptation, the family cohesion subscale consists of seven items measuring support (e.g. ‘In my family we support each other’) and shared values (e.g. ‘In my family we agree on most things’) in the family. The scale comprises seven items rated on a five-point Likert scale ranging from ‘fully disagree’ to ‘fully agree’.

#### Peer problems

Peer problems were measured by the peer problems subscale of the Strengths and Difficulties Questionnaire (SDQ) [29,30]. This subscale consists of five items assessing peer related experiences such as having good friends, being liked by others and being bullied. The items are rated on a three-point Likert scale: ‘not true’, ‘partially true’, ‘completely true’. A previous study from youth@hordaland found adequate psychometric properties of the five-factor solution of the SDQ [31].

### Statistical analysis

The association between parental separation and school dropout was analysed using logistic regressions analyses with a sequential covariate addition. The first model assessed the crude association between parental separation and school dropout. In the subsequent models we examined whether gender, parental education, log EDI, health complaints, family cohesion, and peer relationship problems attenuated this association, by entering these covariates separately in each model. Finally, we tested a fully specified model where all covariates were entered simultaneously.

Gender was controlled for as there were more girls than boys who reported to have separated parents, and as boys have a higher dropout rate from secondary education than girls [32].

Next, to disentangle and quantify the contributions of each covariate in explaining the association between parental separation and school dropout, we conducted a Fairlie decomposition analysis on the fully adjusted model. Fairlie decomposition is an extension of the Blinder–Oaxaca decomposition that quantifies the separate contributions of group differences on binary outcomes [33].

Missing data due to item non-response was fairly low, ranging from 0% (sex) to 5.49% (family cohesion). Listwise deletion was used in all regression analyses.

All analyses were conducted using Stata 17 [34], including the implementation of the Blinder–Oaxaca decomposition procedure [35]. The results from the decomposition analysis are presented visually as a figure created in R using the ggplot2-package [36].

## Results

The sample consisted of slightly more female adolescents than male. On average, adolescents with separated parents had less favourable socioeconomic conditions than those with non-separated parents, with significantly lower parental educational qualifications and lower EDI. Adolescents with separated parents also reported higher levels of health complaints and peer problems, and lower levels of family cohesion than peers from non-separated two-parent families (see Table I for details). Of adolescents with separated parents 21% had not completed upper secondary school within five years, compared with 12% among adolescents with non-separated parents.

**Table I.** Descriptive characteristics of the sample.

	Separated parents *N*=2530	Non-separated parents *N*=5793	*p*-value
	*n* (%)	*n* (%)	
Sex			0.002
Female	1438 (56.4)	3065 (52.8)	
Male	1113 (43.6)	2774 (47.2)	
Parental education^a^			0.001
Basic	197 (7.8)	241 (4.2)	
Intermediate	1215 (48.0)	2304 (39.8)	
High	829 (32.8)	2212 (38.2)	
Advanced	288 (11.4)	1022 (17.66)	
Unknown	1 (0.0)	14 (0.2)	
EDI, mean (SD)	302.5 (169.9)	370.6 (242.1)	<0.001
Health complaints, mean (SD)	5.77 (5.07)	4.69 (4.61)	<0.001
Family cohesion, mean (SD]	3.66 (0.91)	3.93 (0.83)	<0.001
Peer problems, mean (SD)	1.90 (1.67)	1.67 (1.59)	<0.001

*p*-values were derived from Chi square tests and *t*-tests. EDI = equivalized disposable income in 1000 Norwegian Kroner.

a Based on the International Standard Classification of Education (for a full description, see Methods section).

Table II presents the results from the logistic regression analyses. In the crude model, adolescents with separated parents had more than twice the odds (OR = 2.16, 95% confidence interval (CI): 1.90–2.45) of school dropout compared with peers with non-separated parents. Across the model specifications, the higher odds of school dropout only slightly attenuated, and more in the models accounting for parental education and income than in the models accounting for health complaints, peer problems and family cohesion (see Table II). In the fully adjusted model considering all covariates simultaneously, the OR for school dropout was reduced to adjusted OR = 1.72 (95% CI: 1.50–2.00).

**Table II.** The crude and adjusted effects of parental separation on school dropout.

	Model 1 (crude)	Model 1+ sex	Model 1+ parental education	Model 1+ income	Model 1+ health complaints	Model 1+ family cohesion	Model 1+ peer relationship problems	Fully adjusted model
	OR (95% CI)	OR (95% CI)	OR (95% CI)	OR (95% CI)	OR (95% CI)	OR (95% CI)	OR (95% CI)	OR (95% CI)
Parental separation								
No	1	1	1	1	1	1	1	1
Yes	2.16 (1.90–2.45)	2.20 (1.93–2.49)	1.95 (1.71–2.22)	1.96 (1.72–2.24)	2.04 (1.80–2.32)	2.01 (1.76–2.29)	2.07 (1.82–2.36)	1.72 (1.50–1.98)

OR: odds ratio; CI: confidence interval

**Figure 1.**
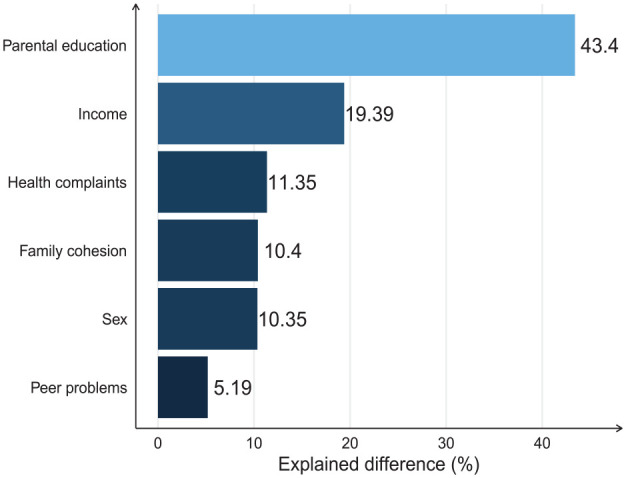
Proportion of the explained difference in school dropout by parental separation accounted for by the covariates.

Results from the decomposition analysis are shown in Figure 1. Overall, the fully adjusted model accounted for 31.17 % of the difference in odds of school dropout between the groups. Of these 31.17 %, parental education accounted for most of the explained difference (43.4 %), followed by EDI (19.4 %). Family cohesion and health complaints accounted for a smaller part of the difference in odds (10–11%), whereas peer problems accounted for only about 5%.

## Discussion

Combining survey and registry data in a large study of Norwegian adolescents, we found that adolescents with separated parents had a twofold higher odds of school dropout compared with peers in a non-separated two-parent family. Socioeconomic characteristics, and particularly parental education, accounted for most of the explained differences between the groups captured by our covariates, whereas less was explained by family cohesion, health complaints and peer problems.

Higher odds of school dropout for youth with separated parents aligns with previous international [1] and Norwegian studies [6] indicating that parental separation is associated with poorer academic outcomes in adolescence. Fewer studies have specifically assessed the link between parental separation and school dropout. Still, our results corroborate two studies from the USA [9,10] finding a lower odds of school dropout for adolescents in non-divorced families compared with those with divorced parents, which suggests that parental separation is associated with a higher odds of school dropout also among Norwegian adolescents.

Accounting for parental education and income, reported health complaints, family cohesion and peer problems reduced the odds of school dropout for adolescents with separated parents. The decomposition analysis suggested that socioeconomic characteristics, and particularly lower parental education qualifications among adolescents with separated parents, were prominent factors in understanding the association between parental separation and school dropout. Parental education is usually established before adults have had the time to create a family or separate from their partner. Further, lower parental educational levels are associated with a higher risk of separation [16] and poorer academic achievement among adolescents [13]. Thus, our results indicate that part of the higher odds of school dropout among adolescents with separated parents could stem from selection based on parental education. In addition to the genetic transfer of academic abilities [13], less educated parents tend to have lower academic expectations and monitor their children’s academic progress less than more educated peers [14], which could be part of the explanation of the higher odds of dropout among youth with separated parents.

EDI accounted for about 20% of the explained difference in school dropout between the groups. Low income may affect adolescents’ academic progress through fewer environmental opportunities and less parental investment in their academic success [15]. As with parental education, income may serve as a selection mechanism, as low income is a predictor for both parental separation and lower academic achievement among youth. Income could also be a mechanism, as parental separation often leads to fewer economic resources [17], and poor financial situation is associated with a higher risk of school dropout [12]. By relying on a static measure of disposable income, we were not able to test the relative merits of these two pathways. Still, our findings suggest that the on average lower household income in families with separated parents explains some of the higher odds of school dropout in this group compared with those in two-parent families. This finding provides some support to the economic deprivation perspective of divorce stating that family economy is one of the components that influences the link between parental separation and academic outcomes among children and youth [37].

Family cohesion, health complaints and peer problems accounted for a smaller share of the explained difference in school dropout between the groups. Thus, although adolescents with separated parents reported more health complaints and peer problems, and less family cohesion than peers in two-parent families, the individual contribution of each of these factors was small compared with parental education and income. It is still worth noting that if viewed combined, these factors accounted for roughly 27% of the explained difference in odds between the groups. Thus, as noted by others [11], multiple factors are likely needed to understand the link between experiences of parental divorce and outcomes in adolescence, even though the individual contribution of each factor may be small.

Our results indicate that these factors account for some but certainly not all of the association between parental separation and school dropout. On the one hand, this suggests that the association between parental separation and school dropout was robust to adjustments of several theoretically relevant variables that might either confound or explain this association. On the other hand, this highlights that other unmeasured factors are likely important to further understand the link between parental separation and school dropout. A recent meta-analysis identified several risk factors for school dropout, including physical and mental health problems, adverse childhood experiences, family and parenting problems, and problems at or with school such as peer problems, learning difficulties or negative attitudes toward school [38]. Although our covariates tap into some of these constructs, more nuanced measures within these domains could have given further insights into the association between parental separation and school dropout.

## Strengths and limitations

A key strength of the present study was the high-quality register-based data on school dropout, parental education and income that was linked to a large survey-based study of a well-defined cohort of older adolescents. The study also used measures of health complaints, peer problems and family cohesion, which all have been found to have adequate psychometric properties on the current study sample. Another strength and a novel contribution was the use of the Fairlie post-regression decomposition, which allowed a detailed analysis of factors potentially accounting for differences in dropout between adolescents with and adolescents without separated parents.

The results from the study should be interpreted in consideration of some limitations. First, the response rate of the entire study was 53%, and the sample size was further reduced due to item non-response. A previous study found that the mean GPA of participants was similar to regional and national averages [39]. Still, the sample was skewed towards higher socioeconomic status when compared with official statistics. Hence, attrition could affect the generalizability of the results. Another main limitation was that all covariates were static measures, and most were measured at the same time. Thus, we cannot infer whether the higher levels of health complaints and peer problems, and lower levels of family cohesion and disposable income among youth in separated parents were a consequence of or have been present before parental separation.

## Conclusions

The present study found that adolescents with separated parents had more than twice the odds of school dropout compared with peers in non-separated two-parent families. This association only partly attenuated after adjustments of objective measures of parental education and income, and self-reported measures of health complaints, family cohesion and peer problems. Thus, adolescents with separated parents are an at-risk group of dropping out from secondary education in Norway. All covariates combined accounted for about 31% of the explained difference in school dropout between the groups. Parental education followed by household income accounted for most of this explained difference. As parental education is often established before parents have had time to have children and separate, our results suggest that some of the higher odds of school dropout among youth with separated parents may stem from selection based on parental education. The findings of the present study further highlight that considering multiple factors is likely needed to better understand the relationship between parental separation and school dropout in adolescence.
